# Hydrogen Sulfide Ameliorated High Choline-Induced Cardiac Dysfunction by Inhibiting cGAS-STING-NLRP3 Inflammasome Pathway

**DOI:** 10.1155/2022/1392896

**Published:** 2022-07-22

**Authors:** Lu Bai, Jing Dai, Yuxuan Xia, Kaichuan He, Hongmei Xue, Qi Guo, Danyang Tian, Lin Xiao, Xiangjian Zhang, Xu Teng, Yuming Wu, Sheng Jin

**Affiliations:** ^1^Department of Physiology, Hebei Medical University, Hebei 050017, China; ^2^Department of Clinical Diagnostics, Hebei Medical University, Hebei 050017, China; ^3^Hebei Collaborative Innovation Center for Cardio-Cerebrovascular Disease, 050017 Hebei, China

## Abstract

Although it is an essential nutrient, high choline intake directly or indirectly via its metabolite is associated with increased risk of cardiovascular disease, the mechanism of which remains to be elucidated. The present study was performed to investigate whether hydrogen sulfide (H_2_S) was involved in high choline-induced cardiac dysfunction and explore the potential mechanisms. We found that ejection fraction (EF) and fractional shortening (FS), the indicators of cardiac function measured by echocardiography, were significantly decreased in mice fed a diet containing 1.3% choline for 4 months as compared to the control, while applying 3,3-dimethyl-1-butanol (DMB) to suppress trimethylamine N-oxide (TMAO, a metabolite of choline) generation ameliorated the cardiac function. Subsequently, we found that feeding choline or TMAO significantly increased the protein levels of cyclic GMP-AMP (cGAMP) synthase (cGAS), stimulator of interferon genes (STING), NOD-like receptor protein 3 (NLRP3), caspase-1, and interleukin-1*β* (IL-1*β*) as compared to the control, which indicated the activation of cGAS-STING-NLRP3 inflammasome axis. Moreover, the protein expression of cystathionine *γ*-lyase (CSE), the main enzyme for H_2_S production in the cardiovascular system, was significantly increased after dietary supplementation with choline, but the plasma H_2_S levels were significantly decreased. To observe the effect of endogenous H_2_S, CSE knockout (KO) mice were used, and we found that the EF, FS, and plasma H_2_S levels in WT mice were significantly decreased after dietary supplementation with choline, while there was no difference between CSE KO + control and CSE KO + choline group. To observe the effect of exogenous H_2_S, mice were intraperitoneally injected with sodium hydrosulfide (NaHS, a H_2_S donor) for 4 months, and we found that NaHS improved the cardiac function and reduced the protein levels of cGAS, STING, NLRP3, caspase-1, and IL-1*β* in mice receiving dietary choline. In conclusion, our studies revealed that high choline diet decreased plasma H_2_S levels and induced cardiac dysfunction via cGAS-STING-NLRP3 inflammasome axis while H_2_S treatment could restore the cardiac function by inhibiting cGAS-STING-NLRP3 inflammasome axis.

## 1. Introduction

Choline is an essential bioactive micronutrient abundant in egg yolk, red meat, fish, dairy products, and soybean. Although it can be formed de novo by methylation of phosphatidylethanolamine, additional dietary intake of choline is also required or else will develop a deficiency state [1]. Because of its wide-ranging roles in biological processes including cholinergic neurotransmission, lipid transport, membrane phospholipids synthesis, and methyl group metabolism, inadequate intake or abnormal metabolism of choline can lead to neurological disorders, cancers, and cardiovascular disease, which can be cured clinically with choline [2–4]. However, an analysis of a large prospective cohort showed high choline intake was associated with increased risk of cardiometabolic mortality in racially diverse populations [5]. And a growing body of preclinical studies highlighted that high choline intake directly or indirectly via its metabolites such as trimethylamine N-oxide (TMAO) had been to a higher risk of heart disease. For example, Organ et al. reported that choline diet and its derived metabolite, TMAO, exacerbated pressure overload-induced heart failure [6]. Another study found that high-choline diet aggravated cardiac dysfunction, fibrosis, and inflammation in a mouse model of heart failure with preserved ejection fraction [7]. Nevertheless, the potential mechanism of high choline-induced cardiac dysfunction remains to be elucidated.

Hydrogen sulfide (H_2_S) is a colorless, water-soluble, and corrosive gas with a characteristic odor of rotten eggs and was traditionally known as an environmental pollutant which is toxic to humans at high concentrations [8]. However, it was not until the pioneering work of Abe and Kimura in 1996 that H_2_S was truly considered to be an endogenous gasotransmitter alongside carbon monoxide and nitric oxide [9]. In mammalian cells, H_2_S is biosynthesized mainly from L-cysteine and/or L-homocysteine by three endogenous enzymes: cystathionine *β*-synthase (CBS), cystathionine *γ*-lyase (CSE), and 3-mercaptopyruvate sulfurtransferase (3-MST) coupled with cysteine aminotransferase. The tissue distribution of these H_2_S-producing enzymes is different: CBS is predominantly expressed in the central nervous system, whereas CSE is mainly present in the cardiovascular system, and 3-MST is found primarily in the brain and erythrocytes [10, 11]. Numerous studies have shown that physiological concentration of H_2_S plays a fundamental role in the cardiovascular system by regulating the biological functions and maintaining homeostasis [12, 13]. Conversely, lack of endogenous H_2_S was detrimental and contributed to various cardiovascular diseases including atherosclerosis, hypertension, myocardial infarction, and heart failure [14–16]. However, whether high choline-induced cardiac dysfunction was associated with the changes in H_2_S concentration has not previously been evaluated.

With this in mind, the aim of present study was to investigate whether H_2_S was involved in high choline-induced cardiac dysfunction and explore the potential mechanisms.

## 2. Material and Methods

### 2.1. Animals and Treatments

All animal experimentals were performed according to the Guide for the Care and Use of Laboratory Animals published by the US National Institutes of Health (NIH Publication, 8th Edition, 2011) and approved by the Ethics Committee for Laboratory Animals Care and Use of Hebei Medical University. Male C57BL/6 J mice were provided from Vital River Laboratories (Beijing, China). CSE knockout (CSE KO) mice with C57BL/6 J genetic bases and its homozygote wild-type (WT) mice were bred from CSE heterozygous mice which were kindly provided as gifts by Professor Yichun Zhu (Fudan University, Shanghai, China). Mice were housed in plastic cages with 12 h light/12 h dark cycles at 22-24°C with 60% humidity and *ad libitum* access to standard rat chow and sterile tap water.

In order to observe the effect of choline, male C57BL/6 J mice were randomly divided into 2 groups: control group and choline group. The mice in the choline group were given a chow diet supplemented with 1.3% choline (Beijing Keao Xieli Feed Co., Ltd., Beijing, China) for 4 months, and the mice in the control group were given a regular chow diet for the same period.

In order to observe the effect of 3,3-dimethyl-1-butanol (DMB, the TMA lyase inhibitors), male C57BL/6 J mice were randomly divided into 3 groups: control group, choline group, and choline + DMB group. The mice in the choline group and choline + DMB group were given a chow diet supplemented with 1.3% choline for 4 months. The mice in the choline + DMB group were fed with 1.3% DMB (Aladdin Biochemical Technology Co., Ltd., Shanghai, China) in the drinking water for 4 months.

In order to observe the effect of TMAO, male C57BL/6 J mice were randomly divided into 2 groups: control group and TMAO group. The mice in the TMAO group were fed with 1.3% TMAO (Aladdin Biochemical Technology Co., Ltd., Shanghai, China) in the drinking water for 2 months.

In order to observe the effect of exogenous H_2_S, male C57BL/6 J mice were randomly divided into 3 groups: control group, choline group, and choline + sodium hydrosulfide (NaHS, a H_2_S donor) group. The mice in the choline group and choline + NaHS group were given a chow diet supplemented with 1.3% choline for 4 months. The mice in the choline + NaHS group were intraperitoneally injected with NaHS (100 *μ*mol/kg/day, Sigma-Aldrich Ltd., St. Louis., USA) for 4 months.

In order to observe the effect of endogenous H_2_S, male CSE KO and WT mice were randomly divided into 4 groups: WT + control group, WT + choline group, CSE KO + control group, and CSE KO + choline group. The mice in the WT + choline group and CSE KO + choline group were given a chow diet supplemented with 1.3% choline for 4 months.

### 2.2. Echocardiography

At the end of the experiment, mice were anesthetized with 1% isoflurane, and the cardiac function was evaluated by using a VisualSonics Vevo 2100 system (FUJIFILM VisualSonics Inc., Toronto, Canada) as described in our previous research [17]. M-mode images of the left ventricle were recorded, and three consecutive cardiac cycles were selected to measure left ventricular ejection fraction and fractional shortening (LVEF and LVFS). And then, the heart was harvested and stored at -80°C until assay. Plasma was separated from the blood after centrifugation at 3500 rpm for 10 min and stored at -80°C until assay.

### 2.3. Measurement of H_2_S Concentration in Plasma

The H_2_S levels in plasma were measured according to the previously study [18]. Briefly, 30 *μ*L of plasma was mixed with 80 *μ*L monobromobimane (MBB, Sigma-Aldrich Ltd., St. Louis., USA) and 10 *μ*L 0.1% ammonia with shaking for 1 h at room temperature for derivatization of sulfide, which called sulfide-dibimane. The reaction was then terminated with 10 *μ*L 20% formic acid and centrifuged at 15000 × g for 10 min. The supernatants were stored at -80°C before the measurement of H_2_S levels using liquid chromatography coupled with tandem mass spectrometry.

### 2.4. Western Blot Analysis

The protein expressions in myocardial tissue were evaluated by western blotting according to the previously study [19]. Frozen heart tissues were homogenized with ice-cold radio immunoprecipitation assay (RIPA) lysis buffer. Proteins were extracted and quantified by the bicinchoninic acid (BCA) method. Equal amount of protein samples was separated on 10% sodium dodecyl sulfate-polyacrylamide gel electrophoresis (SDS-PAGE) gels and transferred to polyvinylidene fluoride membranes. The membranes were blocked with 5% nonfat milk for 1 h and incubated with primary antibodies that recognized CSE (1 : 1000, Santa Cruz Biotechnology, the United States), cyclic GMP-AMP (cGAMP) synthase (cGAS, 1 : 1000, Proteintech Biotechnology, the United States), stimulator of interferon genes (STING, 1 : 1000, Proteintech Biotechnology, the United States), NOD-like receptor protein 3 (NLRP3, 1 : 1000, Proteintech Biotechnology, the United States), caspase-1 (1 : 1000, Proteintech Biotechnology, the United States), interleukin-1*β* (IL-1*β*, 1 : 1000, Proteintech Biotechnology, the United States), and GAPDH (1 : 5000, Proteintech Biotechnology, the United States) at 4°C overnight. Then, the membranes were incubated with horseradish peroxidase-conjugated secondary antibodies for 1 h after washing with TBST. Specific bands were detected with SuperSignal West Pico Chemiluminescent Substrate (Thermo, Scientific-Pierce, Waltham, the United States). The band intensity was quantified by ImageJ software.

### 2.5. Statistical Analysis

The experimental data were presented as mean ± SEM and statistical significance assessed in SPSS (SPSS 13.0, Inc., Chicago, the United States) using independent t-test to compare values between two groups and one-way ANOVA followed by least significant difference t-test to compare values between multiple groups. P < 0.05 was considered statistically significant.

## 3. Results

### 3.1. Dietary Choline Induced Cardiac Dysfunction in Mice

**Figure 1:**
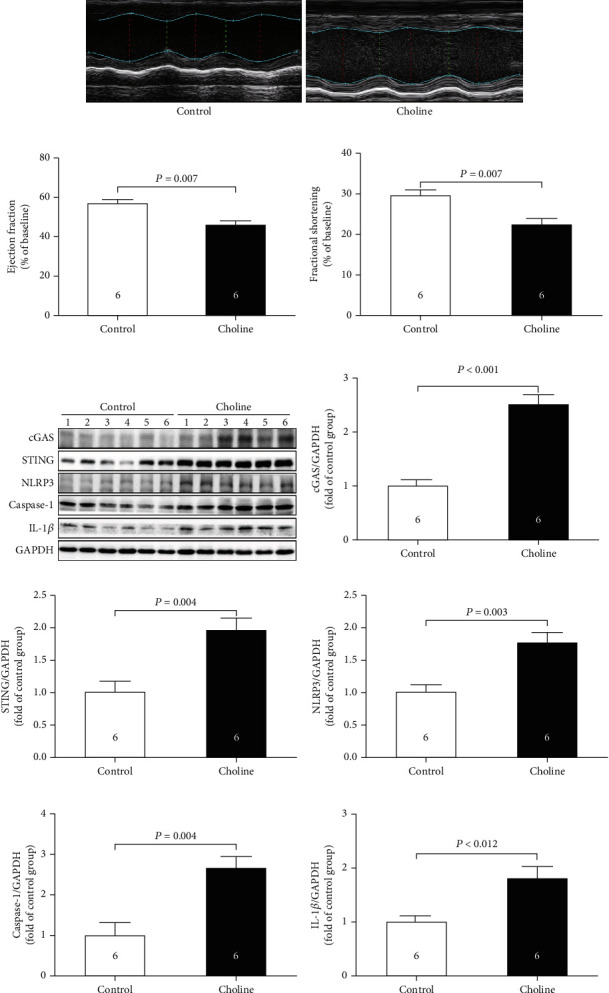
Dietary choline induced cardiac dysfunction in mice. (a) Representative M-mode images. (b) The changes of left ventricular ejection fraction (LVEF) after dietary supplementation with choline. (c) The changes of left ventricular fractional shortening (LVFS) after dietary supplementation with choline. (d)–(i) Representative western blots and quantitative analysis for cGAS, STING, NLRP3, caspase-1, and IL-1*β* protein expression in heart tissues after dietary supplementation with choline. Results are expressed as mean ± SEM. A *P* of <0.05 was considered significant.

As was shown in Figures 1(a)–1(c), EF and FS, the indicators of cardiac function measured by echocardiography, were significantly decreased in mice fed a diet containing 1.3% choline as compared to the control. To better understand the mechanism of the action of dietary choline, we quantified the protein expression of cGAS-STING-NLRP3 inflammasome axis in the heart (Figures 1(d)–1(i)) and found that choline significantly increased the protein levels of cGAS, STING, NLRP3, caspase-1, and IL-1*β* as compared to the control.

### 3.2. Dietary Choline Induced Cardiac Dysfunction by Generating TMAO in Mice

**Figure 2:**
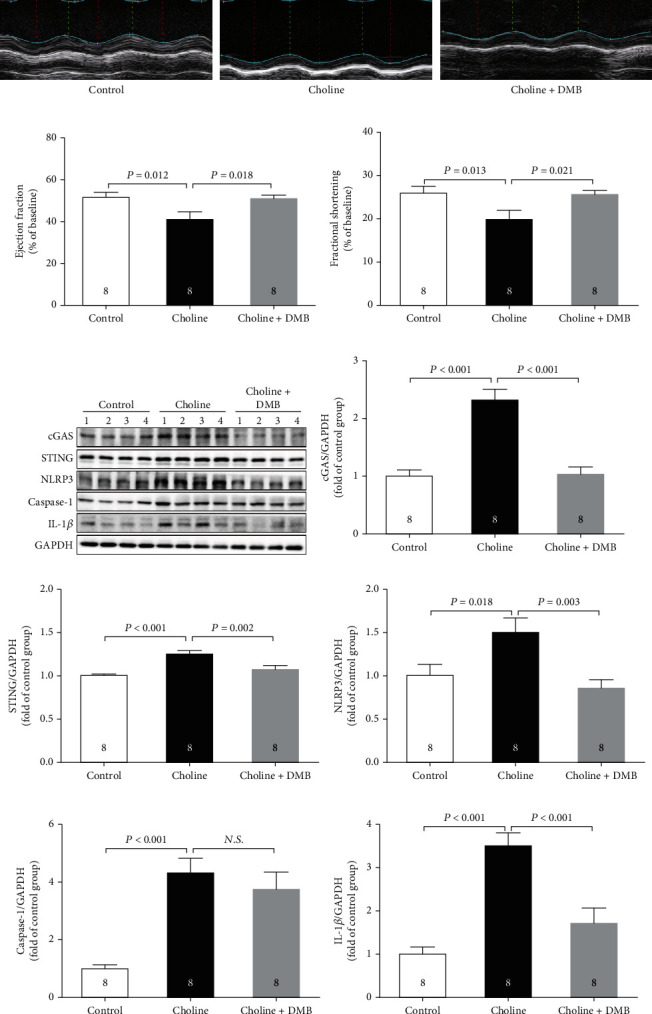
Dietary choline induced cardiac dysfunction by generating TMAO in mice. (a) Representative M-mode images. (b) The changes of left ventricular ejection fraction (LVEF) after DMB supplementation. (c) The changes of left ventricular fractional shortening (LVFS) after DMB supplementation. (d)–(i) Representative western blots and quantitative analysis for cGAS, STING, NLRP3, caspase-1, and IL-1*β* protein expression in heart tissues after DMB supplementation. Results are expressed as mean ± SEM. A *P* of <0.05 was considered significant.

**Figure 3:**
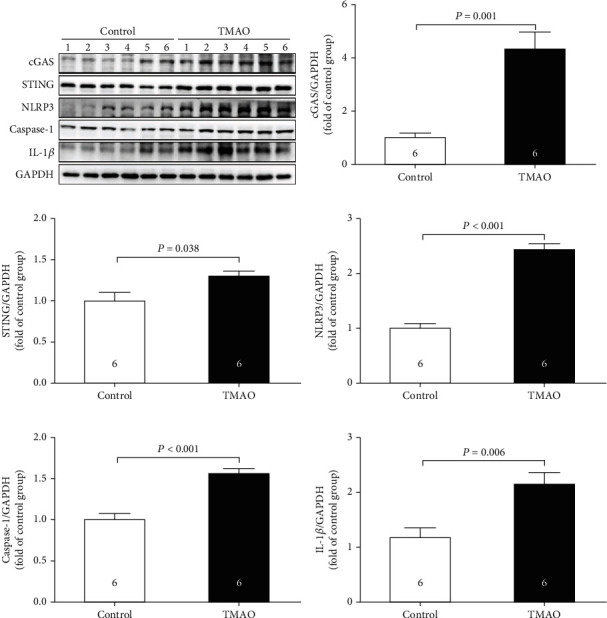
Dietary TMAO upregulated the protein expression of cGAS-STING-NLRP3 inflammasome axis. (a)–(f) Representative western blots and quantitative analysis for cGAS, STING, NLRP3, caspase-1, and IL-1*β* protein expression in heart tissues. Results are expressed as mean ± SEM. A *P* of <0.05 was considered significant.

To explore whether TMAO produced by choline degradation was involved in choline-induced cardiac dysfunction, DMB, a structural analog of choline, was used to inhibit TMAO formation. As was shown in Figure 2(a)–2(c), addition of DMB in the drinking water substantially ameliorated EF and FS as compared to the Choline group. DMB also markedly inhibited the choline diet-induced increase in the protein levels of cGAS, STING, NLRP3, caspase-1, and IL-1*β* (Figures 2(d)–2(i)). In addition, the protein levels of cGAS, STING, NLRP3, caspase-1, and IL-1*β* were significantly increased in mice receiving dietary TMAO as compared to the control (Figures 3(a)–3(f)).

### 3.3. Dietary Choline Inhibited the Endogenous Production of H_2_S

**Figure 4:**
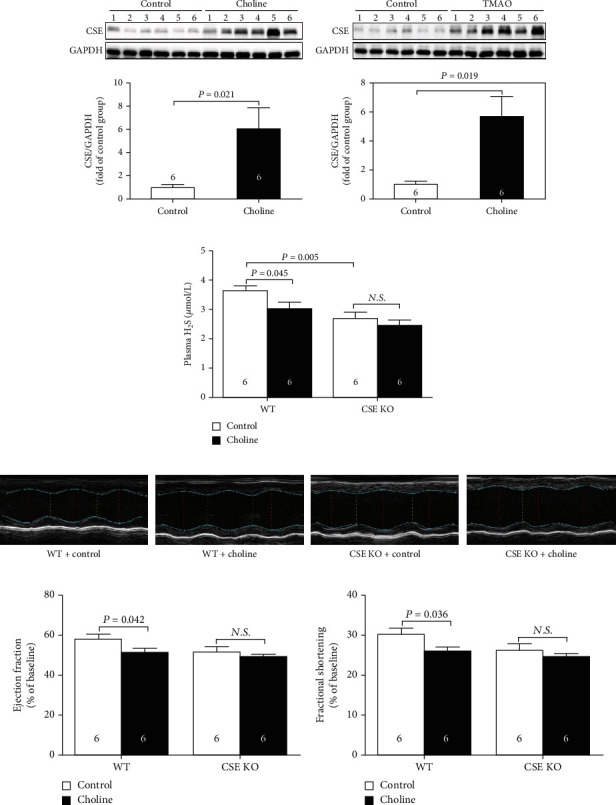
Dietary choline inhibited the endogenous production of H_2_S. (a) Representative western blots and quantitative analysis for CSE protein expression in heart tissues after dietary supplementation with choline. (b) Representative western blots and quantitative analysis for CSE protein expression in heart tissues after dietary supplementation with TMAO. (c) Plasma H_2_S levels in CSE KO mice. (d) Representative M-mode images in CSE KO mice. (e) The changes of left ventricular ejection fraction (LVEF) in CSE KO mice. (f) The changes of left ventricular fractional shortening (LVFS) in CSE KO mice. Results are expressed as mean ± SEM. A *P* of <0.05 was considered significant.

As was shown in Figures 4(a) and 4(b), the protein expressions of CSE, the main enzyme for H_2_S production in the cardiovascular system, were significantly increased after dietary supplementation with choline or TMAO, which indicated that endogenous H_2_S was involved in choline-induced cardiac dysfunction. So, WT and CSE KO mice were fed with choline. As was shown in Figure 4(c), the plasma H_2_S levels in WT mice were significantly decreased after dietary supplementation with choline, while there was no difference in the plasma H_2_S levels between CSE KO + control and CSE KO + choline group. EF and FS were significantly decreased in the WT mice fed with choline, but there was also no significant difference in EF and FS between CSE KO + control and CSE KO + choline group (Figures 4(d)–4(f)).

### 3.4. Exogenous H_2_S Improved Choline-Induced Cardiac Dysfunction

**Figure 5:**
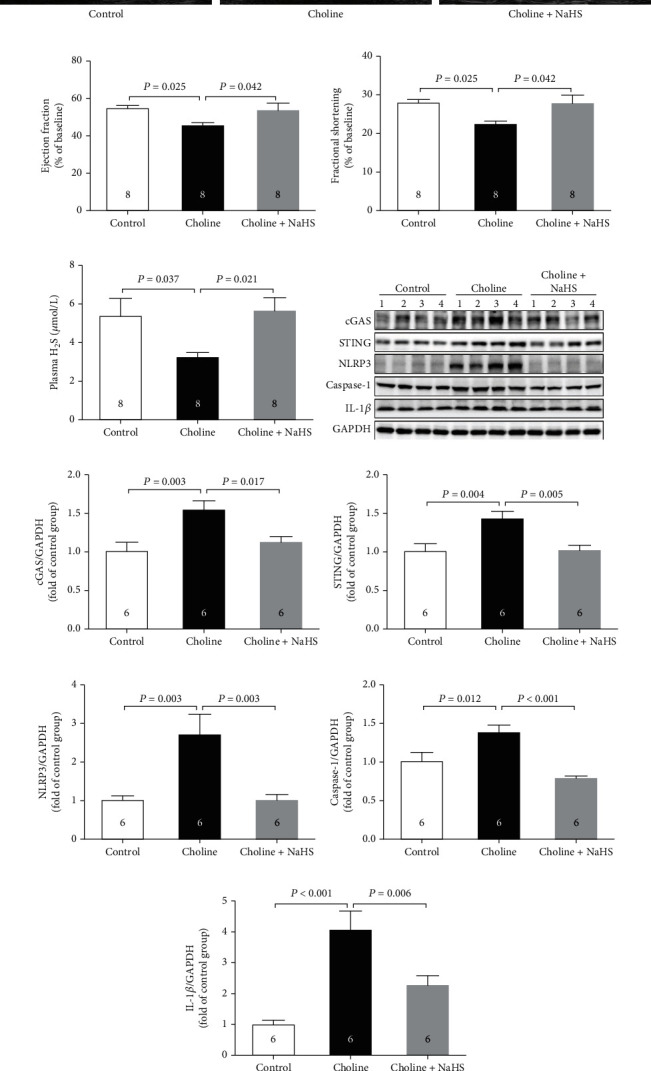
Exogenous H_2_S improved choline induced-cardiac dysfunction. (a) Representative M-mode images. (b) The changes of left ventricular ejection fraction (LVEF) after NaHS treatment. (c) The changes of left ventricular fractional shortening (LVFS) after NaHS treatment. (d) Plasma H_2_S levels after NaHS treatment. (e)–(j) Representative western blots and quantitative analysis for cGAS, STING, NLRP3, caspase-1, and IL-1*β* protein expression in heart tissues after NaHS treatment. Results are expressed as mean ± SEM. A *P* of <0.05 was considered significant.

As was shown in Figures 5(a)–5(c), compared with the choline group, both EF and FS were significantly increased in the choline + NaHS group; meanwhile, the plasma H_2_S levels were also markedly increased in the choline + NaHS group (Figure 5(d)). In addition, NaHS reduced the protein levels of cGAS, STING, NLRP3, caspase-1, and IL-1*β* in mice receiving dietary choline (Figure 5(e)–5(j)).

## 4. Discussion

In the present study, we found that high choline diet induced cardiac dysfunction via cGAS-STING-NLRP3 inflammasome axis while H_2_S treatment could restore the cardiac function by inhibiting cGAS-STING-NLRP3 inflammasome axis.

Although it played vital physiological roles in the development and function of the cardiovascular system as an essential nutrient, emerging evidence implicated that higher dietary intakes of choline were also associated with increased risk of increased risk of acute myocardial infarction (MI) in patients with stable angina pectoris [20]. Moreover, a high-choline diet was shown to exacerbate the cardiac function and cardiac fibrosis of MI mice through accelerating the transformation of fibroblasts into myofibroblasts [21]. Choline in the diet can be metabolized to trimethylamine (TMA) by the intestinal microorganisms. After being absorbed into the blood, TMA enters the liver and is oxidized to TMAO which is involved in the onset and development of cardiovascular disease. It was reported that two weeks of TMAO injection significantly induced cardiac hypertrophy and fibrosis in rats [22]. In the present study, the cardiac function represented by EF and FS was significantly decreased in mice after 4 months of 1.3% choline feeding, while applying DMB to suppress TMAO generation improved the cardiac function. Subsequently, we found that feeding choline or TMAO promoted NLRP3 inflammasome formation as well as caspase-1 and IL-1*β* activation. The NLRP3 inflammasome is an intracellular protein complex activated upon tissue injury. Once activated, it can trigger and amplify sterile inflammatory responses by activating and releasing IL-1*β*, which has been reported to involve in the pathophysiology cardiovascular disease [23, 24]. In line with our findings, one study reported that choline uptake in bone-marrow-derived macrophages regulated activation of the NLRP3 inflammasome, whereas impaired choline uptake and phosphorylation reduced NLRP3 inflammasome activation and inhibited IL-1*β* production [25]. Another study reported that TMAO aggravated doxorubicin-induced mouse cardiac fibrosis through activation of the NLRP3 inflammasome [26]. Moreover, Wu et al. reported that either a high-choline diet or TMAO enhanced the allogenic graft-versus-host (GVH) reaction which was mediated by NLRP3 inflammasome activation-induced macrophage polarization, whereas DMB reversed choline-induced GVH disease severity [27].

Given the chemical and structural diversity of NLRP3-activating stimuli, it is unlikely that those stimuli directly bind to and activated NLRP3. Instead, NLRP3 is likely to sense a common cellular signal induced in response to NLRP3 activators. Multiple molecular or cellular events including K^+^ efflux, Ca^2+^ signaling, reactive oxygen species, mitochondrial dysfunction, and lysosomal damage, were involved in the activation of NLRP3 inflammasome assembly [28, 29]. Recently, it was found that mitochondrial DNA (mtDNA) which was released into the cytoplasm played an important role in the activation of the inflammasome [30]. The DNA sensor cGAS interacted with mtDNA and generated the second messenger cGAMP, which trigger the cGAS-STING-NLRP3 pathway to activate inflammasome response [31]. In the present study, we found that feeding choline or TMAO increased the protein expression of cGAS and STING, while DMB markedly inhibited the choline diet-induced increase in the protein levels of cGAS and STING, which indicated that feeding choline or TMAO activated the cGAS-STING pathway. Although there was no direct evidence that choline or TMAO promoted mtDNA release, it was confirmed that TMAO altered mitochondrial energy metabolism [32] and enhanced the mitochondrial impairments [33], which might induce mtDNA release to trigger the cGAS-STING -NLRP3 pathway [34].

As the third endogenous signaling gasotransmitter, H_2_S participates in a wide spectrum of physiological processes in the body including regulating mitochondrial function. Although higher concentrations inhibit the electron transport chain [35], lower concentrations promote mitochondrial biogenesis and function [36, 37]. However, to the best of our knowledge, there are currently no studies exploring the link between H_2_S and the cGAS-STING pathway. In the present study, we found that dietary choline significantly decreased the plasma H_2_S levels, while application of H_2_S donor, NaHS, significantly increased plasma H_2_S levels and inhibited cGAS-STING pathway. However, how H_2_S inhibited the cGAS-STING pathway still needed to be further elucidated. In addition, we also found that NLRP3 inflammasome activation was inhibited by NaHS, which was generally consistent with our previous studies and others' reports. Our previous studies clarified that H_2_S improved hypertension-associated endothelial dysfunction [38] or attenuated lipopolysaccharide-induced acute kidney injury [39] by inhibiting NLRP3 inflammasome. H_2_S was also reported to protect against dextran sulfate sodium-induced colitis [40] or paraquat-induced acute liver injury [41] by inhibiting NLRP3 inflammasome.

Moreover, we found that the protein expressions of CSE, the main enzyme for H_2_S production in the cardiovascular system, were significantly increased after dietary supplementation with choline or TMAO, but the plasma H_2_S levels were significantly decreased. Our results were consistent with previous studies in which a significant decrease in H_2_S bioavailability was observed in the plasma, aorta, or myocardial tissue but a higher CSE expression in aorta or myocardial tissue [42, 43]. It was reported that the CSE could function as an inducible H_2_S generating enzyme, whose expression was upregulated in cells by a range of stimuli including endoplasmic reticulum stress, oxidative stress, nutrient deprivation, and hyperhomocysteinemia [44]. The elevated CSE protein expression could be explained as a compensatory mechanism; although, this compensation did not increase plasma H_2_S levels, which was due to the accelerated H_2_S metabolism by choline or TMAO induced oxidative stress [45, 46]. On the other hand, CSE produced H_2_S at the steady-state low intracellular Ca^2+^ concentrations in cells [47], whereas choline or TMAO could increase Ca^2+^ influx and/or Ca^2+^ release from intracellular stores to inhibit CSE activity and suppress H_2_S generation [48, 49]. To further confirm the compensatory increased expression of CSE, CSE KO mice were used, and we found that the plasma H_2_S levels in WT mice were significantly decreased after dietary supplementation with choline, while there was no difference in the plasma H_2_S levels between CSE KO + control and CSE KO + choline group. Our finding meant that choline or TMAO relied on CSE protein to regulate H_2_S levels, but the exact mechanisms remained unclear.

Several limitations of the present study should be noted. Firstly, direct evidence of how choline, TMAO, or H_2_S regulated the cGAS-STING pathway needs to be found. Secondly, how choline or TMAO affected the expression of CSE or other H_2_S generating enzyme need to be further explored in future studies.

In conclusion, our studies revealed that high choline diet decreased plasma H_2_S levels and induced cardiac dysfunction via cGAS-STING-NLRP3 inflammasome axis while H_2_S treatment could restore the cardiac function by inhibiting cGAS-STING-NLRP3 inflammasome axis.

## Data Availability

All data supported the findings of this study can be available from the corresponding author upon reasonable request.
